# Emotion-specific nocebo effects: an fMRI study

**DOI:** 10.1007/s11682-017-9675-1

**Published:** 2017-02-16

**Authors:** Anne Schienle, Carina Höfler, Sonja Übel, Albert Wabnegger

**Affiliations:** Clinical Psychology, University of Graz, BioTechMedGraz, Universitätsplatz 2/DG, 8010 Graz, Austria

**Keywords:** Nocebo, Disgust, Olfactory system, Functional magnetic resonance imaging, Psychophysiological interaction

## Abstract

The neurobiological mechanisms of nocebos are still poorly understood. Thirty-eight women participated in a ‘smell study’ using functional magnetic resonance imaging. They were presented with an odorless stimulus (distilled water) together with the verbal suggestion that this fluid has an aversive odor which enhances disgust feelings. The nocebo was presented while the participants viewed disgusting, fear-inducing, and neutral images. Participants’ affective and neuronal responses during nocebo administration were compared with those in a control condition without nocebo. Twenty-nine women (76%) reported perceiving a slightly unpleasant and arousing odor. These ‘nocebo responders’ experienced increased disgust during the presentation of disgusting images in combination with the nocebo and showed enhanced left orbitofrontal cortex (OFC) activation. It has been suggested that the OFC is involved in the generation of placebo/nocebo-related expectations and appraisals. This region showed increased functional connectivity with areas involved in interoception (insula), autobiographical memories (hippocampus), and odor imagery (piriform cortex) during nocebo administration. The nocebo-induced change in brain activation was restricted to the disgust condition. Implications for psychotherapy are discussed.

## Introduction

The neurobiological mechanisms underlying placebo effects, particularly those relating to placebo analgesia, have been extensively studied (for a review see Wager and Atlas 2015). It has been shown that the administration of a physically and pharmacologically inert drug, device or other type of intervention reduces the level of pain experienced by patients, if they believe they are receiving an active treatment. This positive expectation, which can be elicited by means of learning experiences, verbal suggestions or other contextual cues, is accompanied by specific changes in brain activation. Very consistently these changes included increased activation of the ventromedial/dorsolateral prefrontal cortex, and the orbitofrontal cortex (OFC). The localized prefrontal activity is thought to be involved in the generation of placebo-related positive expectations and appraisals (Wager and Atlas 2015). Flaten et al. (2011) have argued that such expectations lead to pain relief via a decrease in negative emotions. Reduced activation during placebo analgesia can be found in those brain regions involved in interoceptive awareness and pain processing (e.g., insula, anterior cingulate cortex, periaqueductal gray).

The opposite phenomenon, nocebo hyperalgesia, has attracted less research attention. In general, nocebo effects include the occurrence of negative symptoms, the worsening of a condition, and the prevention of improvement after the administration of an inert substance or a sham treatment. Nocebo effects are a consequence of negative expectations by the patients (for a review see Häuser et al. 2012). The neuronal systems, which are activated by ‘nocebo expectations’ are still poorly understood, and it is a matter of debate whether placebo and nocebo effects share a common brain network or whether they are characterized by distinct neuronal features. Schmid et al. (2013) demonstrated that deceptive information regarding an intravenous drug treatment resulted in increased pain experience and insula activation during visceral stimulation (painful rectal distensions). In a subsequent study by the authors (Schmid et al. 2015), nocebo-responders (who had reported pain sensitization) displayed increased activation in the insula, the somatosensory cortex and the amygdala during painful rectal stimulation. In addition, the insula showed increased functional connectivity with the midcingulate cortex as a function of negative expectations. Consequently, the insula was identified as a central hub for the transmission of placebo as well as nocebo effects.

Freeman et al. (2015) directly compared the effects of placebo and nocebo interventions. They administered a heat pain stimulus together with an inert cream, which was explained as being either a pain-reducing or pain-increasing substance. The expectation of increased pain activated the insula, the OFC, and the periaqueductal gray, whereas the placebo recruited the striatum. These findings point to a completely separated placebo/ nocebo network.

The majority of previous nocebo neuroimaging studies have investigated nocebo effects in the context of pain (e.g., Kong et al. 2008). Only rarely have other response systems been analyzed via fMRI, e.g., nocebo effects on itch (Napadow et al. 2015). The present nocebo study aimed at changing disgust experiences and was based on previous observations of a ‘disgust placebo’ (Schienle et al. 2014a, b). The authors of that study presented their disgust-prone participants with affective images (disgusting, fear-eliciting, neutral) and administered a placebo pill together with the verbal suggestion that this was an effective ‘anti-nausea’ (disgust-reducing) medication. This intervention very effectively diminished feelings of disgust: the intensity of experienced revulsion was almost halved by the placebo. This effect was accompanied by reduced insula activation. Psychophysiological interaction (PPI) analyses revealed that placebo administration altered connectivity in a network consisting of the insula, the amygdala, and the OFC. Interestingly, the placebo did not evoke neuronal changes during the presentation of fear-inducing pictures. These fear-inducing pictures served as an ‘affective’ control condition in the experiment. Thus, the placebo provoked emotion-specific effects in accordance with the disgust-reducing suggestion.

A recent fMRI study (Schienle et al. 2017) directly compared neuronal effects of explicit and implicit disgust regulation (cognitive reappraisal vs. placebo administration) in women with average disgust proneness. Relative to the passive viewing of disgust images, both reappraisal and placebo treatment significantly reduced self-reported disgust. In addition, both regulation strategies were associated with similar connectivity patterns. Placebo and reappraisal increased the coupling between the OFC and the amygdala relative to passive viewing. The OFC is involved in cue–outcome learning and reward/punishment expectations (Wager and Atlas 2015).

In contrast to these previous studies, the present experiment focused on disgust-enhancing expectations. The participants received the misinformation that an administered substance (distilled water) would have a slightly aversive, disgusting odor. Investigations with similar instructions have been conducted previously. Jaén and Dalton (2014) exposed asthmatic patients to a harmless odorant (rose scent). Half of the participants were told that the odor might cause respiratory problems, whereas the other half received the information that the odor was therapeutic. The patients in the nocebo condition showed increased airway inflammation.

The design for the present nocebo study followed on from a previous placebo experiment (Schienle et al. 2014a) regarding the presentation of affective images from three categories (disgust, fear, neutral). It was hypothesized that a ‘disgust nocebo’ would specifically increase experienced disgust during the presentation of disgust images, while the other conditions would not be affected. This should be accompanied by increased brain activation as well as functional connectivity in a network consisting of key regions involved in placebo/nocebo effects (e.g., insula, OFC).

In an exploratory approach, it was investigated whether nocebo responses in the context of disgust processing, would be associated with trait disgust. This personality characteristic refers to individual differences in sensitivity to disgust (Schienle et al. 2002a). Disgust-prone subjects experience disgust more frequently and more intensely across various situations. This may make them particularly susceptible for disgust-related nocebo suggestions.

## Method

### Sample

Thirty-eight women were invited to a ‘smell study’. The cover story stated that they would be presented with a disgusting odor of very low intensity in order to test olfactory sensitivity. All participants were recruited via announcements at the university campus and had a high school diploma. The sample had been restricted to women because there are significant sex differences in disgust proneness (Schienle et al. 2002a). Exclusion criteria consisted of mental disorders, medication, and somatic problems as assessed by the Brief Symptom Inventory (Derogatis 1993). Written informed consent was obtained from all subjects. The study was conducted in accordance with the Declaration of Helsinki and had been approved by the ethics committee of the University of Graz.

From the 38 participants, nine were excluded from further analysis because they did not respond to the nocebo suggestion at all (‘non-responders’). Data from the remaining 29 right-handed, non-smoking, healthy women with a mean age of 22.31 years (SD = 2.95) were analyzed.

### Materials

#### Nocebo

**Fig. 1:**
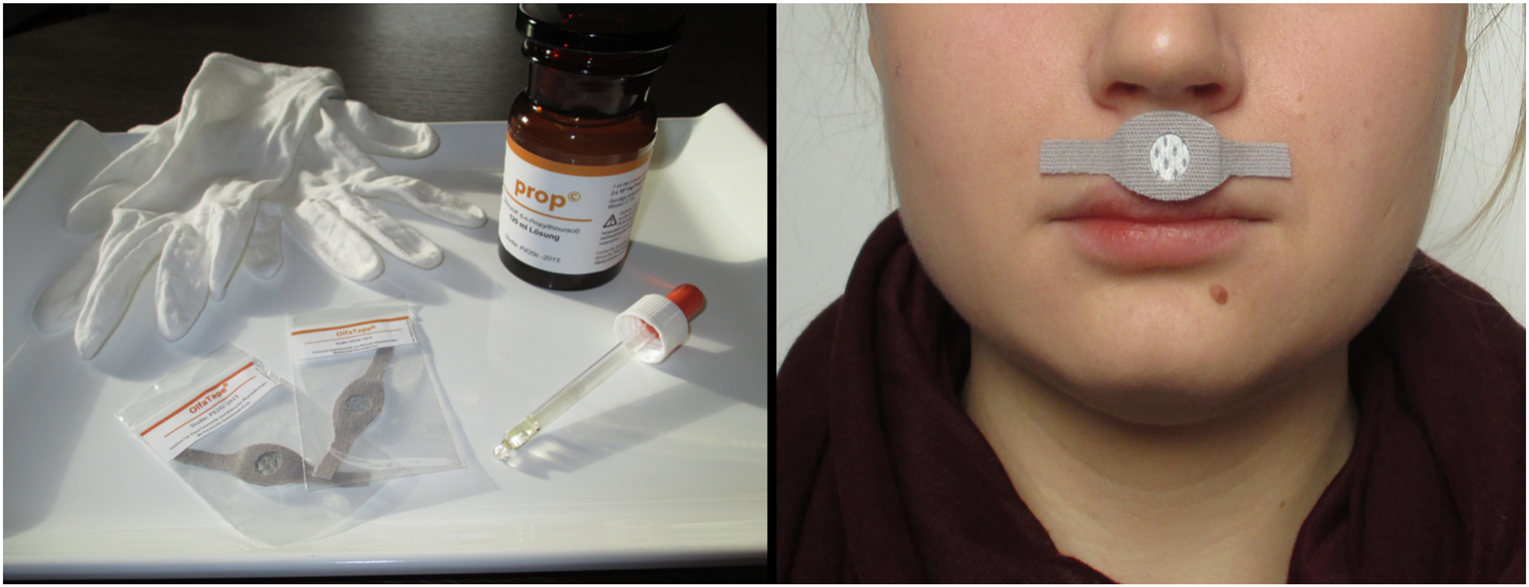
Nocebo administration

We administered 0.2 ml distilled water with green food coloring to an odorless tape (labeled as ‘Olfatape’) attached beneath the nose. The size of the tape was 4 cm × 1 cm (see Fig. 1). During the application of Olfatape the experimenter wore odorless gloves to prevent the participants from detecting smells from the experimenter’s hand. The participants were instructed that the fluid was a chemical (6-n-Propylthiouracil; PROP) with an aversive odor (‘smells like sour milk, rancid butter, vomit’). This fluid would be presented in a very low dosage (just above the olfactory threshold). In addition, it was mentioned that a pilot study had already shown that this substance is able to increase feelings of disgust. The findings of this study were illustrated with a poster attached to the wall of the lab. The subjects were asked to rate the distilled water according to experienced valence (1 = ‘very pleasant‘; 9 = ‘very unpleasant‘) and odor intensity (1 = ‘does not smell at all‘, 9 = ‘smells very intensely‘). None-responding had been defined by an intensity rating of ‘1’ (*n* = 9).

#### Disgust proneness

The participants answered the Questionnaire for the Assessment of Disgust Proneness (QADP; Schienle et al. 2002a). This personality trait refers to the general tendency of a person to experience disgust across different situations. The self-report measure consists of 37 items that have to be judged on 5-point scales (0 = ‘not disgusting’, 4 = ‘very disgusting’), e.g. ‘you smell vomit’. The Cronbach’s alpha of the total scale is .90. The mean QADP score was 2.09 (SE = 0.10), which did not differ from the score of the construction sample (*p* = .40).

#### Pictures and design

A total of 90 affective images from the categories Disgust (e.g., rotten food, poor hygiene), Fear (e.g., acts of violence, dangerous predators), and Neutral (pixelated disgust and fear images) were administered. Each category consisted of 30 different pictures, which had been divided into two parallel sets of 15 pictures each. The images were selected from the International Affective Picture System (IAPS; Lang et al. 2008) and from a validated set of the authors (Schienle et al. 2002b).^1^ The images were presented for 4 s each in pseudorandomized order to avoid that more than two pictures of the same category were presented in succession. The inter-stimulus intervals (presentation of a fixation cross) varied between 3.5 and 8 s. Each image was shown twice within each condition (= 30 events per picture category).

The experiment consisted of a nocebo condition and a control condition (without nocebo) during which the participants passively viewed affective images (30 Disgust, 30 Fear, 30 Neutral). The two conditions were separated by a short break (approximately 3 min), during which Olfatape was either attached or removed. The sequence of the two conditions was balanced across the participants. Further, the sequence of the two parallel picture set was counterbalanced across the conditions.

At the end of the nocebo and the control condition the subjects were presented with three slides depicting an overview of the previously viewed pictures representing an affective category (Disgust, Fear, Neutral). They were asked to rate experienced arousal, disgust, and fear on 9-point-Likert scales (9 = very arousing, disgusting, fear-eliciting). This rating procedure was the same as in the previous ‘disgust placebo’ study (Schienle et al. 2014a).

### fMRI recording

The fMRI session was conducted with a 3 T scanner (Skyra, Siemens, Erlangen, Germany). Functional runs were acquired using an echo-planar imaging protocol (number of slices: 35, descending, flip angle =90°, slice thickness: 3 mm; matrix: 64 × 64 mm; TE = 30 ms; TR = 2290 ms; FoV: 192 mm; in-plane resolution =3 × 3 × 3 mm). All analyses were conducted using SPM12 (Wellcome Department of Cognitive Neurology, London). Three volumes from the beginning of the time series were discarded to account for saturation effects.

First, acquisition timing was accounted in a slice timing step followed by motion-correction in the realignment and unwarping step. Afterwards, individuals T1 images were coregistered to their functional data and were segmented into grey matter (GM), white matter (WM) and cerebrospinal fluid. To increase inter-subject alignment individual images of GM and WM were registered in a ‘Fast Diffeomorphic Registration Algorithm’ (DARTEL) to the IXI550 template implemented in the VBM 8 toolbox. Resulting individual DARTEL flow fields were used to normalize slice-timed, realigned and unwarped functional images to MNI-space (3 mm isotropic voxels). Finally, for smoothing a Gaussian kernel of 6 mm was applied. Vectors for each event of interest (picture onset) were compiled and entered into the design matrix to model event-related responses by the canonical hemodynamic response function in the first level stage. Data were high pass filtered (128 s). As realignment and unwarping already model the B0*motion interaction, motion parameters were not included in the first level analysis as regressors. Temporal sphericity was controlled by an AR(1) process with consecutive prewhitening of the data.

### fMRI analyses

For the fMRI data, planned t-contrasts (e.g., Disgust > Neutral; Disgust > Fear) were computed to compare the two conditions (Nocebo, Control). Exploratory whole-brain voxel intensity tests were conducted as well as region of interest (ROI) analyses for the amygdala (number of voxels: left (L) 64, right (R) 75), the insula (L: 569, R: 527), the hippocampus (L: 283, R: 262), and the OFC (L: 1522, R: 1552). These regions were selected based on previous findings on disgust and nocebo effects (e.g., Schienle et al. 2002a, b, Wager and Atlas 2015). Additional ROIs involved in odor perception/ imagery were chosen (piriform cortex (L: 415, R: 467), entorhinal cortex (L: 81, R: 94)).

Further, multiple regression analyses were conducted to correlate QADP scores with ROI activation. The used ROI masks were taken from the AAL atlas (Tzourio-Mazoyer et al. 2002) and were created with the WFU PickAtlas (WFU Pickatlas v2.4; Wake Forest University School of Medicine). The AAL atlas was created by the anatomical parcellation of a single-subject T1-weighted volume with manually drawn regions of interest. For all analyses the height threshold was set at *p* < 0.001 uncorrected for at least five contiguous voxels. Results were small volume-corrected and considered significant if *p* < .05 (corrected for family-wise error (FWE)).

Moreover, psychophysiological interaction (PPI) analyses (Friston et al. 1997) were conducted to investigate functional connectivity in the two conditions (Nocebo, Control). PPI assesses the extent to which an experimental factor modulates the connectivity of one brain region with others, in terms of condition-specific covariation in residuals. Given specific seed regions, PPI identifies voxel activation that covaries differentially with the seed region as a function of an experimental factor. For each participant, a PPI analysis was performed by setting up a design matrix containing three columns of variables: the first regressor, the physiological variable, represents the time series of activity taken from the seed region by taking the first eigenvariate of the corresponding mask. The second regressor, the psychological variable, represents the condition type (e.g. the contrast Nocebo_Disgust > Nocebo_Neutral). The PPI variable (PPI term) represents the third regressor, which was computed as the element by element product of the deconvolved extracted time series of the selected seed region and a vector coding for the effect of task. Subject-specific contrast images were then entered into a paired t-test analysis in order to explore connectivity (Disgust > Neutral) for the contrasts Nocebo > Control and Control > Nocebo.

The left OFC was defined as seed region, because here significant activation for the contrast Nocebo > Control: Disgust > Neutral occurred (nocebo activation). For the PPI analyses the height threshold was set at *p* < 0.05 uncorrected for at least five contiguous voxels. Results were small volume corrected and considered significant if *p* < .05 (corrected for family-wise error (FWE)). The ROIs were the same as for the planned t-tests.

## Results

### Self-report

#### Nocebo ratings

The nocebo responders gave the following ratings for the distilled water: M(intensity) = 4.07 (SE = .25; range: 3–7), and M(valence) = 5.79 (SE = .21).

#### Picture ratings

The ratings were analyzed with repeated measures ANOVAs (SPSS; version 22) with the within-subject factors Picture Category (Disgust, Fear, Neutral) and Condition (Nocebo, Control). We report η2p (partial eta^2^) as effect size measure as well as Greenhouse-Geisser epsilon (ɛ). Significant effects were followed up with post-hoc t-tests with Bonferroni correction (cut off: alpha/3 = .017).

The conducted ANOVA for the disgust ratings of the images revealed significant effects for Picture Category (F(2,27) = 275.61, *p* < .001, ɛ = .911, η2p = .953) and the interaction Picture Category x Condition (F(2,27) = 4.30, *p* = .044, ɛ = .800, η2p = .242). The main effect for Condition was marginally significant F(1,28) = 3.31, *p* = .08, η2p = .106). The conducted post-hoc t-tests showed that the nocebo administration increased experienced disgust for the Disgust images (*p* = .001), but not for Fear and Neutral images (p’s > .53).

For the fear ratings, only the effect for Picture Category reached statistical significance (F(2,27) = 59.07, *p* < .001, ɛ = .963, η2p = .814). Fear images received higher fear ratings than Disgust and Neutral pictures (all p’s < .01).

The conducted ANOVA for the arousal ratings revealed significant main effects for Condition (F(1,28) = 7.93, *p* = .009, η2p = .221) and Picture Category (F(2,27) = 39.51, *p* <. 001, ɛ = .984, η2p = .721). The conducted post hoc t-tests showed that the nocebo administration increased arousal ratings (*p* = .009) and that Disgust pictures and Fear pictures were perceived as more arousing than Neutral pictures (both p’s < .001). Arousal ratings for Disgust and Fear pictures did not differ from each other (*p* = .24). The ratings are displayed in Table 1. The introduction of an additional factor Sequence (nocebo-control vs. control-nocebo) to the analyses of variance produced no significant main or interaction effects (all p’s > .15).

**Table 1:** Affective picture ratings (means and standard errors)

Affective ratings	Control M (SE)	Nocebo M (SE)
Disgust pictures		
Disgust	6.72 (.28)	7.38 (.25)
Fear	2.69 (.31)	3.24 (.35)
Arousal	4.93 (.33)	5.48 (.41)
Fear pictures		
Disgust	2.55 (.27)	2.76 (.27)
Fear	5.62 (.36)	6.17 (.39)
Arousal	4.52 (.34)	5.07 (.33)
Neutral pictures		
Disgust	1.52 (.26)	1.48 (.20)
Fear	1.17 (.09)	1.38 (.22)
Arousal	2.00 (.26)	2.52 (.37)

#### Correlation analyses

Disgust proneness (QADP) was neither correlated with nocebo-related changes in disgust experience (difference: Nocebo – Control) for Disgust images nor with intensity and valence ratings for the nocebo (all p’s > .16). Nocebo-related changes in disgust experience were not correlated with nocebo ratings (both p’s > .62).

### Brain imaging

The Disgust pictures (contrast: Disgust > Neutral) elicited activation in the selected regions of interest (insula, amygdala, OFC, piriform cortex, hippocampus) during the control condition as well as during nocebo administration (Table 2). These ROIs were also recruited during fear processing (contrast: Fear > Neutral).

**Table 2:** Results of the planned t-contrasts for the regions of interests

Region	H	X	Y	Z	t	P(FWE)	Cluster size
Control: Disgust > Neutral							
Insula	R	27	15	-21	6.95	<.001	164
	L	-33	24	0	7.88	<.001	192
Amygdala	R	24	-6	-15	10.62	<.001	62
	L	-24	-9	-12	11.36	<.001	45
Hippocampus	R	24	-6	-18	9.70	<.001	58
	L	-21	-9	-12	11.39	<.001	71
Orbitofrontal cortex	R	30	33	-12	10.41	<.001	140
	L	-30	33	-9	8.40	<.001	151
Piriform cortex	R	36	-36	-15	11.65	<.001	70
	L	-24	-6	-18	10.67	<.001	47
Nocebo: Disgust > Neutral							
Insula	R	33	27	0	8.05	<.001	54
	L	-33	27	3	7.19	<.001	130
Amygdala	R	24	-3	-18	9.72	<.001	62
	L	-21	-3	-18	7.94	<.001	40
Hippocampus	R	24	-6	-18	8.09	<.001	28
	L	-18	-6	-12	6.74	<.001	44
Orbitofrontal cortex	R	27	33	-15	8.81	<.001	103
	L	-33	33	-15	9.20	<.001	125
Piriform cortex	R	24	-3	-18	9.72	<.001	60
	L	-21	-3	-18	7.94	<.001	40
Control: Fear > Neutral							
Insula	R	27	18	-18	7.23	<.001	73
	L	-33	24	0	6.46	<.001	80
Amygdala	R	21	-6	-12	11.15	<.001	66
	L	-30	-3	-18	8.36	<.001	42
Hippocampus	R	18	-6	-12	9.46	<.001	118
	L	-21	-12	-12	8.18	<.001	97
Orbitofrontal cortex	R	33	33	-15	10.50	<.001	169
	L	-36	27	-15	7.18	<.001	147
Piriform cortex	R	36	-36	-15	11.52	<.001	100
	L	-27	-42	-9	8.89	<.001	50
Nocebo: Fear > Neutral							
Insula	R	33	24	0	5.48	.002	29
	L	-36	24	3	4.86	.007	19
Amygdala	R	30	-3	-21	7.65	<.001	50
	L	-30	-3	-21	6.60	<.001	23
Hippocampus	R	30	-6	-21	6.66	<.001	59
Orbitofrontal cortex	R	36	33	-15	7.23	<.001	109
	L	-3	39	-21	6.02	<.001	57
Piriform cortex	R	36	-39	-12	12.20	<.001	94
	L	-24	-42	-9	9.13	<.001	40
Nocebo > Control: Disgust > Neutral							
Orbitofrontal cortex	L	-18	48	-15	4.78	.018	15

*H* hemisphere, *x,y,z* MNI coordinates, *p(FWE) p*-value (corrected for family-wise error), *cluster size* number of voxels in associated cluster

**Fig. 2:**
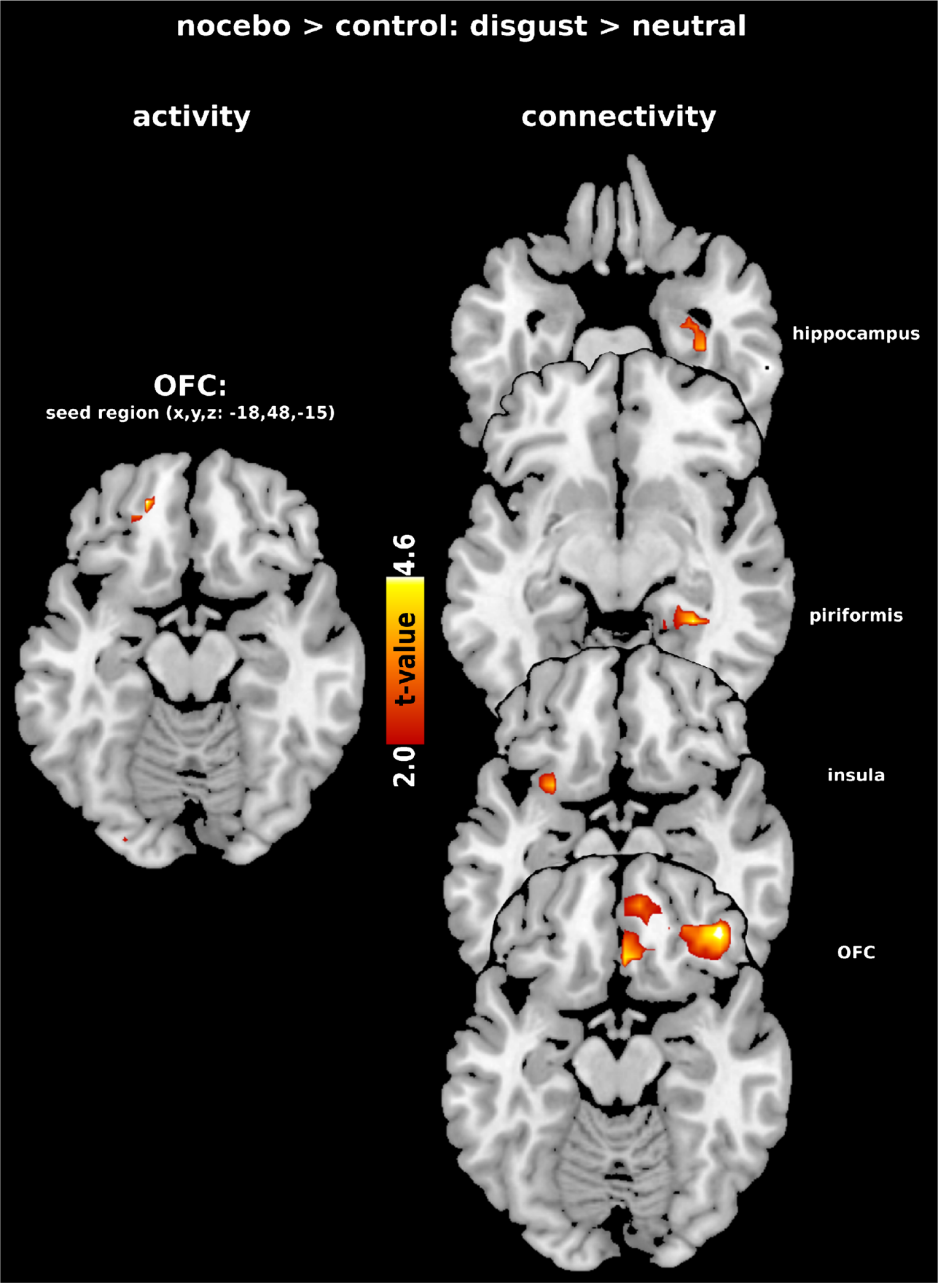
Increased left orbitofrontal cortex (OFC) activation during nocebo administration and increased connectivity with regions of interest (right OFC, hippocampus, piriform cortex, left insula)

Relative to the control condition, the nocebo was associated with increased left OFC activation (contrast: Nocebo > Control: Disgust > Neutral; Fig. 2). The reversed contrast, as well as Fear > Neutral produced no significant effects. A similar result characterized the contrast Disgust > Fear with stronger orbitofrontal activation in the nocebo condition (x,y,z, left: −42,30,24, *t* = 4.36, p(FWE) = 0.025, cluster size =259 voxels). There were no significant whole brain effects.

The regression analysis revealed a positive correlation between QADP scores and orbitofrontal activity in the left (x,y,z: −9,42,-12, *t* = 4.46, p(FWE) = 0.043, cluster size =149 voxels) and right hemisphere (x,y,z: 6,42,-18, *t* = 5.02, p(FWE) = 0.009, cluster size =123 voxels) for the contrast Nocebo > Control: Disgust > Neutral.

For the PPI analysis the left OFC was chosen as the seed, because this area had been activated by the nocebo. The OFC showed enhanced coupling with the right piriform cortex (x,y,z: 30,-39,-9, *t* = 4.51, p(FWE) = 0.011, cluster size =117), the left insula (x,y,z: −27,12,-15, *t* = 3.96, p(FWE) = 0.032, cluster size =20), the right OFC (x,y,z: 42,36,-15, *t* = 4.75, p(FWE) = 0.018, cluster size =318) and the right hippocampus (x,y,z: 36,-18,-24, *t* = 3.83, p(FWE) = 0.015, cluster size =54) in the nocebo condition relative to the control condition (see Fig. 2).

### Discussion

This fMRI study investigated the emotion specificity of nocebo effects. The majority of invited participants (76.3%) reported an odor perception in accordance with the cover story regarding a ‘smell study’. These nocebo responders perceived ‘Olfatape’ as a slightly unpleasant stimulus of mild to moderate intensity. In addition, the nocebo altered the processing of affective images. This was shown in a slight, but statistically significant increase in experienced disgust. The nocebo-related disgust amplification was restricted to the disgust condition and was not present during the viewing of fear-inducing or neutral images. In other words, the nocebo elicited an emotion-specific effect.

It should be noted that compared with previously observed effects relating to a ‘disgust placebo’ (Schienle et al. 2014a, b), where the experienced disgust intensity was almost halved, the nocebo-related increase seen here was relatively small. This however corresponds with the suggestion of an aversive odorant of very low intensity (close to the sensory threshold). A different nocebo type had been used in a pilot study: a nocebo pill labeled as a nausea-inducing medication. This treatment however not only increased disgust but also feelings of anxiety and concerns about possible side effects of this drug. Thus, it was not possible to develop an emotion-specific nocebo with higher intensity based on this method.

The brain imaging data were in line with the self-report data. In the control condition, the disgust images elicited activation in the predefined ROIs, such as the insula, the amygdala, and the OFC, underlining the point that we were able to provoke the target emotions (e.g., Phan et al. 2002).

The nocebo augmented activation of the left OFC. Similar results have been reported before by Freeman et al. (2015). In this study, the application of an inert cream labeled as Capsaicin (an irritant, which produces a burning skin sensation) evoked increased expectancies regarding pain and significant blood flow changes in the OFC. Furthermore, prior studies on placebo analgesia have demonstrated that positive expectations and evaluations were mediated by this frontal area (e.g., Petrovic et al. 2002; Wager and Atlas 2015). Petrovic et al. (2005) have suggested that the lateral OFC participates in a generalized expectancy modulatory network. In line with this are the findings from Sarinopoulos et al. (2006) who presented their subjects with a highly aversive bitter taste. A placebo instruction, that the stimulus would only be moderately bitter, induced OFC activation predicting subsequent attenuation of bilateral insula responses. Thus, the OFC does appear to be a shared brain region of the placebo and nocebo network.

Findings from the connectivity analysis of the present experiment point to a possible nocebo mechanism. During nocebo administration, the left OFC showed enhanced coupling with the right piriform cortex, the left insula, the right OFC and the right hippocampus. Previous research has found the insula and the hippocampus to be most consistently involved in nocebo responses, e.g. during nocebo hyperalgesia (Wager and Atlas 2015; Schmid et al. 2015). Wager and Atlas (2015) conceptualized the placebo/nocebo response as a ‘meaning response’, which is based on expectancies (OFC), interoceptive assessments of one’s own body state (insula), and autobiographical memories (hippocampus). In the present investigation exactly these regions were identified, together with the piriform cortex (PC), which possibly added a specific sensory ingredient (odor illusion) to this meaning response. The PC is involved in odor identification and the assignment of odor valence. It plays an active role, from the sensory to more cognitive aspects of human olfactory perception (for a review see Bensafi 2012). Bensafi et al. (2007) demonstrated that the PC is not only involved in the smelling of an actual odor, but is also recruited during odor imagery. Our nocebo instruction very likely triggered such imagery processes.

The present study has important clinical implications. The neuropsychological processes that mediate affective nocebo effects may be relevant for a wide array of therapeutic approaches, including the treatment of somatic conditions as well as psychotherapy (Benedetti et al. 2007). It is generally accepted that one basic prerequisite for meaningful change in behavior therapy is the elicitation of realistic expectations for treatment (Leutgeb et al. 2009). For example, in the context of exposure therapy for specific phobias (e.g., spiders, blood), patients are told that confrontation with the disorder-relevant stimulus will have adverse effects including the occurrence of intense negative feelings (e.g., fear, disgust). According to our data, this approach might amplify the negative evaluation of the phobic stimulus and may therefore become a roadblock to change. The correlation analysis indicated that disgust-prone women (with high QADP scores) showed enhanced nocebo-related OFC activation. Considering that patients with a blood or spider phobia are also characterized by an elevated proneness to disgust, this finding underlines the clinical relevance of affective nocebo effects (Leutgeb et al. 2009).

The following limitations of our investigation need to be mentioned. Only women were investigated, since they are characterized by greater disgust responsivity (Schienle et al. 2002a, b). This reduced inter-gender variance, but as a consequence the findings cannot be generalized to men. Furthermore, the number of non-responders (*n* = 9) was not sufficient to compare their brain responses with those of the responders. Future studies should include such a group to be able to further deepen our knowledge about affective nocebo responding. Also, the inclusion of a placebo condition would allow to identify specific as well as shared neuronal components of placebo/nocebo responses.

In conclusion, to the best of our knowledge, this is the first fMRI study on affective (disgust-related) nocebo effects. The study demonstrated that a nocebo was able to elicit a new symptom (‘aversive odor’) and to intensify visually induced disgust. Moreover, OFC activation and connectivity were specifically influenced by the nocebo.

## Footnotes

1 The numbers of the IAPS pictures (Lang et al. 1999) were: 9300, 9320, 9570 for disgust and 1300, 3500, 3530, 6212, 6230, 6312, 6350, 6370, 6510, 6540, 6940, 9910, 9921, 0014, 1525, 2690, 6260, 6300, 6313, 6315, 6530, 6571, 9250, 9912, 9920 for fear images. The remaining pictures were taken from a validated picture set from Schienle et al. (2002b).
